# Precision Teaching and Learning Performance in a Blended Learning Environment

**DOI:** 10.3389/fpsyg.2021.631125

**Published:** 2021-02-05

**Authors:** Bin Yin, Chih-Hung Yuan

**Affiliations:** School of Economics and Commerce, University of Electronic Science and Technology of China, Zhongshan Institute, Zhongshan, China

**Keywords:** blended learning, precision teaching, self-efficacy, learning motivation, community of inquiry, learning performance, structural equation modeling

## Abstract

Blended learning has gained increasing popularity in colleges and universities with mixed results. Precision teaching can effectively promote learning performance. The relation between perceived precision teaching (PPT) and the learning performance of college students in a blended learning environment is investigated in this paper. In the research survey is featuring a structural model, 256 college students who attended blended learning courses featuring precision teaching participated. The model results revealed that PPT is directly and positively related to self-efficacy and learning motivation. Self-efficacy and learning motivation are directly and positively related to cognitive, teaching, and social presence. Additionally, cognitive, teaching, and social presence are directly and positively related to learning performance. Therefore, PPT is remotely and indirectly related to learning performance. These findings provide a new perspective for the theoretical study on blended learning performance and provide a realistic reference for precision teaching practice in the blended learning environment.

## Introduction

Blended learning, as a combination of traditional face-to-face learning and online learning, is aimed at creating a learning atmosphere in support of self-oriented learning, and it claims many benefits (Garrison and Kanuka, 2004; Alammary et al., 2014) such as improving learning efficiency, satisfaction, and learning performance. A long-range research survey conducted by the University of Central Florida (UCF) measured the success of tens of thousands of students in face-to-face learning, blended learning, and online learning environments. UCF defines success as obtaining a Grade C, at least. Factors such as the students’ college, gender, and financial means were also considered. The UCF reported that, for each college, blended learning has a higher success rate than face-to-face learning or online learning (Dziuban et al., 2004). Blended learning may also reinforce flexibility (Moskal et al., 2013; Alammary, 2019) while providing more opportunities for students to access higher education and simultaneously allowing the school to have more contact with students (Vaughan, 2007; Erbil, 2020). Blended learning can also allow for greater spatial and temporal flexibility of both teachers and students so that they have the freedom to decide when and where to organize online teaching and learning (Sharpe et al., 2006; King and Arnold, 2012). It also helps to improve cost-effectiveness and resource use rate (Moskal et al., 2013). In comparison with face-to-face teaching, blended teaching is more likely to further cut operating costs down (Vaughan, 2007). By promoting more students to select courses and the use of network facilities, blended learning requests less classroom time than face-to-face courses, and maintains a greater student retention rate than online courses, thus reducing the time required for students to complete the degree (King and Arnold, 2012; Yuan and Wu, 2020). Blended learning has gained increasing popularity in colleges and universities (Alammary et al., 2014; Porter et al., 2014; Ghazal et al., 2018).

Empirical studies on the learning performance of blended learning derive mixed results. Shea and Bidjerano (2014) discovered that senior high school graduates who received some online or remote courses from American community colleges have a greater probability to obtain the certificate than their peers who solely attended classroom teaching. At the University of Granada, the introduction of blended learning into basic accounting courses raised students’ exam results and lowered their dropout rate (Victoria Lopez-Perez et al., 2011). Students seemingly hold a positive attitude toward it. Jones and Chen (2008) reported students’ positive experiences in blended MBA accounting courses. The negative effect of blended learning is reported by other studies (Xu and Jaggars, 2011). Numerous studies further reveal learners’ problems with persistence in online and blended learning environments, which can be evidenced by a greater dropout rate compared with traditional face-to-face teaching (Xenos et al., 2002; Levy, 2007). Thus, it is necessary to set forth the related factors associated with learning performance in blended learning environments (Ramirez-Arellano et al., 2018).

Scholars have discussed many factors related to learning performance, such as the fundamental individual factors of learners, learning motivation, learning performance expectations, learning time investment (Hsu and Hsieh, 2011; Dakduk et al., 2018; Law et al., 2019), the self-regulating learning ability in the learning process (Sabah, 2020), learning attitude (Hsu and Hsieh, 2011), and prior experience of online learning (Lim and Morris, 2009) all predict learning performance. Regarding course factors, course content, structure clarity, course teaching methods, and modes of teaching predict learning performance (So and Brush, 2008; Afacan, 2016; Putri et al., 2019). As for learning support, a perceived learning system serviceability, usability, perceived flexibility, convenience for collaborative learning (Kerzic et al., 2019; Sabah, 2020), interactivity (Blieck et al., 2019), information support and process guidance (Cocquyt et al., 2019), and emotional support (So and Brush, 2008) predict learning performance.

Despite the many efforts made to figure out factors related to learning performance, few studies have paid attention to precision teaching. Precision teaching is defined as a method that monitors students’ ability to access education. By monitoring students’ learning process results, teachers can adjust their teaching and intervention measures to ensure the optimal learning performance of students (Rebecca and Michelle, 2016). Precision teaching may obviously promote learning performance (Datchuk, 2017; Mannion and Griffin, 2018). With the extensive application of information technology in the education industry, teachers can more conveniently monitor students’ learning process results, make decisions on this basis (Bruhn et al., 2020), and timely adjust their teaching intervention measures to promote learning performance. Information technology has laid a good foundation for the effective implementation of precision teaching (Kubina and Yurich, 2012).

This research explores the relation between perceived precision teaching (PPT) and learning performance in blended learning environments. Self-efficacy and learning motivation are both related to learning performance (Bandura and Watts, 1995; Schneider and Preckel, 2017; Law and Geng, 2018). As a major theoretical model concerning blended learning, a community of inquiry (COI), is composed of social, teaching, and cognitive presence which all predict learning performance (Law et al., 2019). To deepen the correlation among PPT, self-efficacy, learning motivation, and COI, questions have been proposed for further research as below:

Q1: How does PPT predict learning performance in blended learning?
Q2: In blended learning, what is the role of self-efficacy, learning motivation, and COI (including social presence, teaching presence, and cognitive presence) in the relation between PPT and learning performance?

To address the aforementioned research questions, the researchers interviewed 256 students who attended blended course learning featuring precision teaching and performed a modeling analysis on the survey results using structural equation modeling (SEM) to conclude the relationship between PPT and learning performance in blended learning environments. The research aims to provide a realistic reference for the development of precision teaching practice for blended learning environments.

Subsequent chapters of the paper first review the relevant literature related to the theoretical foundation, put forward research hypotheses, materials and methods, following a report on the results for further discussion, and finally draw a conclusion and points out the research significance.

## Theoretical Background

### Blended Learning

Accompanying the application of information and network technology, blended learning is becoming more popular in colleges and universities. Apart from the changes in the traditional face-to-face learning mode, blended learning makes full use of the convenience and rich resources of the internet, and combines this with the advantages of traditional learning. It is a new learning mode that reinforces teaching by computer and network online activities in traditional courses (Benbunan-Fich, 2008). The adoption of blended learning symbolizes the restructuring of curriculum design, which is intended for mobilizing students’ initiative in participating in online learning.

Some studies have proven that integrating information technology into the teaching and learning process creates the acquisition of course resource information and the improvement of the learning experience (Bai et al., 2016; Darling-Aduana and Heinrich, 2018; Turvey and Pachler, 2020). Blended learning methods are also able to significantly enhance the learning experience. With a higher course satisfaction rate than traditional classroom teaching (Darling-Aduana and Heinrich, 2018), the learning method makes students more devoted to the learning process (Yildirim, 2005), and thus it is preferred by students for its greater flexibility and convenience (Hogarth, 2010). A blended learning course design is a sophisticated topic that involves numerous factors, among which learning experience is a predominant factor.

### Learning Performance

Learning performance can be defined in different ways. For instance, it can refer to students’ test scores (Ferguson and DeFelice, 2010; Ekwunife-Orakwue and Teng, 2014; Law and Geng, 2019), satisfaction with learning (Ekwunife-Orakwue and Teng, 2014; Yuan et al., 2020), or even their performance logged in the online learning system (Yang et al., 2016). This research adopts the definition of learning performance as described by the Association for Educational Communications and Technology in 2004, which states that learning performance is the ability of a learner to apply the newly acquired knowledge or skills. In essence, it does not solely involve the basic knowledge and skills learned, but the capability to apply them. There are many factors for learning performance (Broadbent, 2017; Li and Tsai, 2017; Li et al., 2018; Wei and Chou, 2020). In blended learning, COI is an essential theoretical model. With a focus on PPT, this research combines the dimensions in COI, such as teaching presence, social presence, and cognitive presence to investigate the relationship with learning performance. The research also examines the link between these dimensions and other learning characteristics.

### Precision Teaching

Originating from America in the 1960s, precision teaching has been propagated in Britain by education psychologists (EPS) since the 1980s (Muncey and Williams, 1981; Raybould and Solity, 1982, 1988). It essentially pertains to a method of teaching that monitors students’ acquisition of basic education skills. Through monitoring students’ learning process results, teachers are capable of adjusting their teaching and intervention measures to ensure the optimal learning performance of students (Rebecca and Michelle, 2016). Precision teaching involves four basic steps: Pinpoint, Record, Change, and Try Again (Kubina and Yurich, 2012).

Precision teaching, as an effective method to reinforce students’ acquisition of basic education skills (Chapman et al., 2005; Gist and Bulla, 2020), can stimulate students’ learning motivation and get students more engaged in learning (Rebecca and Michelle, 2016). Precision teaching resounds to the improvement of learning performance (Datchuk, 2017; Mannion and Griffin, 2018).

### Perceived Precision Teaching

Perceived precision teaching is defined as the learners’ perception of precision teaching during their study, including the perception of the four basic steps, that is, deciding targeted learning goals, recording the learning process, teaching with purpose, and re-examining the attainment of goals (Pinpoint, Record, Change, and Try Again). PPT can be measured by surveying learners (Dibbs et al., 2013; Rebecca and Michelle, 2016).

### Self-Efficacy

Self-efficacy indicates the individual belief in the success of performing a task (Bandura and Adams, 1980), which directly affects an individual’s behavioral motivation in the implementation of a specific activity. Generally speaking, a successful experience consolidates self-efficacy, while recurrent failures damage self-efficacy.

Self-efficacy is often divided into universal self-efficacy and task-based self-efficacy (Sherer et al., 1982), of which the latter is suited to given tasks and situations and is the dominant type of blended learning. Self-efficacy determines the content and essence of learners’ inner imagination about future learning scenes or procedures, and directly or indirectly affects individual psychological momentum while performing learning activities, thereby generating an impact on real-life learning activities and forming strong associations with learning performance (Bandura and Watts, 1995; Chamorro-Premuzic et al., 2010; Schneider and Preckel, 2017).

### Learning Motivation

According to Ford (1992), learning motivation is defined as a psychological mode in pursuit of goals, beliefs, and emotions. Motivation refers to something that encourages, instructs, and maintains behaviors, approving of students’ devotion to a given direction and persistence in it (Fredricks et al., 2004; Reeves, 2006).

Learning motivation is a key factor influencing learning performance. It is not only a decisive factor in learning performance. Actually, it corresponds to a matched motivation in every task to be accomplished (Weiner, 1990). There are two types of learning motivation, namely extrinsic learning motivation and intrinsic learning motivation. In particular, intrinsic motivation is the dominant type of student learning in blended learning, which implies that students motivated by intrinsic motivation have better task accomplishment learning performance than those motivated by extrinsic motivation (Lin et al., 2003). Numerous studies emphasize the importance of motivation, given its effect on learning performance (Law et al., 2010; Law and Breznik, 2017; Law and Geng, 2018).

### Community of Inquiry

**FIGURE 1:**
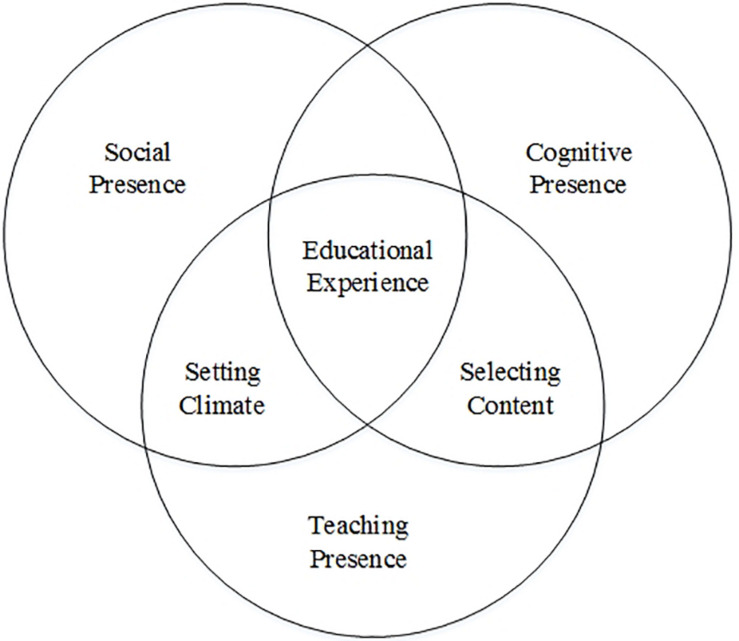
The community of inquiry (Law et al., 2019).

Community of inquiry is widely considered as a model on how members in a society acquire knowledge and solve problems (Garrison et al., 2000). Its core lies in the education experience influenced by the interaction between social, cognitive, and teaching presence. These three types of presence are paramount to the education-oriented COI and can improve or constrain education experience and learning outcomes (Garrison et al., 2000). The framework is shown in Figure 1.

### Cognitive Presence

Cognitive presence is the student’s ability to construct meanings through discussion and reflection in COI, which demonstrates the process of learning and investigation (Garrison, 2011). It entails trigger events, exploration, integration, and reflection, as well as the resolution of learning problems. In the triggering stage, learning tasks can be generated based on doubts about certain knowledge so that students can be prompted into exploration. In the second stage, students can exchange information and engage in critical thinking and investigation in the learning community. In the integration stage, students construct meanings based on the reflection and thoughts from the exploration stage. Finally, students solve their problems in the learning tasks by directly or indirectly using the meanings constructed from the last stage. For the formation of cognitive presence, COI provides an enabling environment to establish and confirm meanings through constant reflection and critical discourse (Garrison et al., 2000).

### Social Presence

Social presence means the ability for individual students to get in touch with the community, conduct meaningful communication in a trustworthy environment, and develop interpersonal relationships by demonstrating individual characteristics (Garrison, 2011). To achieve social presence, the model must afford an open communication environment that is useful in fabricating the favorable relationship between team cohesiveness and individuals. Students may express their thoughts and emotions with a communication method popular in the community (Garrison et al., 2000). Social presence presents a sense of belonging and also a mechanism of free expression to students and sustains team cohesiveness. As a supporting function of cognitive presence, it indirectly promotes the critical thinking process of learners in the community.

### Teaching Presence

Teaching presence is defined as “the design, facilitation, and direction of cognitive and social processes for the purpose of realizing personally meaningful and educationally worthwhile learning outcomes” (p.3) (Anderson et al., 2001). Teaching design involves designing courses and teaching methods and organizing relevant discussions. Facilitation means to create a learning environment that encourages people to share their personal views for effective communication. Direction is to collectively discuss and resolve problems. In essence, teaching presence is the atmosphere of teaching that combines social and cognitive presence effectively and efficiently (Garrison, 2011).

## Research Hypotheses

### Perceived Precision Teaching, Self-Efficacy, and Learning Motivation

There are four steps in precision teaching, including deciding targeted learning goals, recording learning processes, teaching with purpose, and re-examining the attainment of goals (Pinpoint, Record, Change, and Try Again) (Kubina and Yurich, 2012). In an environment of blended learning featuring precision teaching, learners can perceive that there are clear goals in their study, relevant learning materials, and lectures targeting learning problems. Such perceptions can raise the expectation of success in attaining the learning goals, which is likely to ignite learning motivation (Gorges and Kandler, 2012; Cho et al., 2019). When learners clearly perceive the goals to attain and preview the lectures on their own with the online learning system, they tend to be more oriented and purposeful in their studies, so that their self-efficacy is improved to complete learning tasks according to their goals (Law et al., 2010). When they finish previewing the lectures, they proceed to self-testing to see if the goals are attained, which conveniently indicates to the learners the goals achieved and those yet to be achieved. Checking off the goals can motivate the learners and inform them of the unattained goals, which facilitates intentional planning for the next steps and increases self-efficacy for goal attainment (Hong and Park, 2012; Doumen et al., 2014). During lectures, teachers organize teaching and discussion around the problems that have occurred in the learners’ learning process, so that students can perceive “the lectures are exactly about what they themselves do not quite understand,” thus adding to the motivation to invest more efforts into learning (Rebecca and Michelle, 2016). After the lectures, teachers provide tests based on the feedback during lectures and the learning goals. This way, after self-review, in-class learning, and self-testing post-class, students have a better understanding of the content and hence increased learning motivation (Roberts and Norwich, 2010; Rebecca and Michelle, 2016). Therefore, we put forward the following hypotheses:

H1. PPT is positively related to self-efficacy.
H2. PPT is positively related to learning motivation.

### Self-Efficacy and COI

Self-efficacy directly affects individuals’ dynamic psychological processes in the learning performance of specific activities (Wood and Bandura, 1989), decides their behavioral choices, and forces them to prefer the environment or activity that they feel they are most competent in. It may also affect their efforts, meaning that individuals put in more energy as long as they maintain high self-efficacy over an assignment (Paglis and Green, 2010).

In a blended learning environment with precision teaching, teachers outline precise goals and requirements. In this case, students with high self-efficacy actively contact the teachers, discuss with their classmates, and finish the tasks as required. The higher the self-efficacy, the more active the students would be in sharing knowledge and engaging in discussions (Hsu et al., 2007), and thereby, the stronger would be the social presence that they experience. In the process, those with higher self-efficacy tend to make more efforts in learning and have a better perception of the carefully crafted atmosphere for teaching, that is, a better perception of teaching presence (Akyol and Garrison, 2011). In precision teaching, students preview the content and finish self-testing according to their goals before the lectures. During the lectures, teachers organize teaching and discussion based on the problems that have occurred in the self-learning and self-testing, and further test if the goals are achieved after the lectures. Students with higher self-efficacy can immerse better into the entire learning process (Christina, 2000; Park et al., 2012), which would increase the cognitive presence of learners (Celikkaleli, 2014; Niemiec and Lachowicz-Tabaczek, 2015). To examine the influence of self-efficacy in the blended learning environment, the following hypotheses are proposed:

H3. Self-efficacy is positively related to COI.
H3a. Self-efficacy is positively related to cognitive presence.
H3b. Self-efficacy is positively related to social presence.
H3c. Self-efficacy is positively related to teaching presence.

### Learning Motivation and COI

Motivation embodies the in-depth intention of every learner for a direction or goal. Bandura (1996) considers that people commit to their work or task out of personal belief, and learners’ belief in ability usually better reflects motivation than their actual ability. Corresponding to a group of physiological processes, motivation may determine behavioral direction and persistence (Moos and Marroquin, 2010). If a student wants to improve or develop their own ability in a given task or discipline, they usually seek pertinent help or reflects repeatedly.

Although blended learning courses offer flexible and convenient online learning content, students may lack the motivation to complete the tasks (Bennett et al., 2012; Torrisi-Steele and Drew, 2013), and suffer from procrastination (Cerezo et al., 2017). Motivated students have a strong connection with their teachers and endeavor to establish social relations with their peers (Patrick et al., 2007; Wentzel et al., 2010; Law and Geng, 2018). Such quality would improve the social presence in learning. By actively engaging in online and classroom learning, these students can effectively become accustomed to a digitalized learning environment and cope with learning problems, resulting in a better teaching presence (Zimmergembeck and Locke, 2007; Manganelli et al., 2019). Highly motivated students may make full use of the advantages of precision teaching to learn and explore, and eventually experience a better cognitive presence (Manganelli et al., 2019). The following hypotheses are thus presented:

H4. Learning motivation is positively related to COI.
H4a. Learning motivation is positively related to cognitive presence.
H4b. Learning motivation is positively related to social presence.
H4c. Learning motivation is positively related to teaching presence.

### COI and Learning Performance

In a COI course, students take more personal responsibilities and share multiple viewpoints more actively. Through vigorous online interaction, students and teachers together can create a COI environment with social, cognitive, and teaching presence (Akyol et al., 2011; Kozan and Richardson, 2014). This implies that extensive participation of teachers and their clarity in course design would affect students’ perceived learning (Swan, 2001). In some teaching and learning environments, in an engineering course, for instance, teachers’ performance plays a decisive role in learning performance, outweighing social and cognitive presence in importance (Szeto, 2015). Students’ involvement in discussions, their motivation, and cooperation with others can influence their learning and performance (Chong, 1998; Swan, 2001; Davies and Graff, 2005). In the research on the effects of cohesion and interaction on team performance among students, social presence is closely related to learning outcomes (Arbaugh, 2005; Williams et al., 2006). The COI framework suggests an approach to creating collaboration and meaningful learning experiences, which can improve or undermine education experiences and learning outcomes (Garrison et al., 2000). Three types of presence are thus predictive of learning performance (Arbaugh, 2008; Choy and Quek, 2016). Hence, we propose the following hypotheses:

H5. COI is positively related to learning performance.
H5a. Cognitive presence is positively related to learning performance.
H5b. Social presence is positively related to learning performance.
H5c. Teaching presence is positively related to learning performance.

**FIGURE 2:**
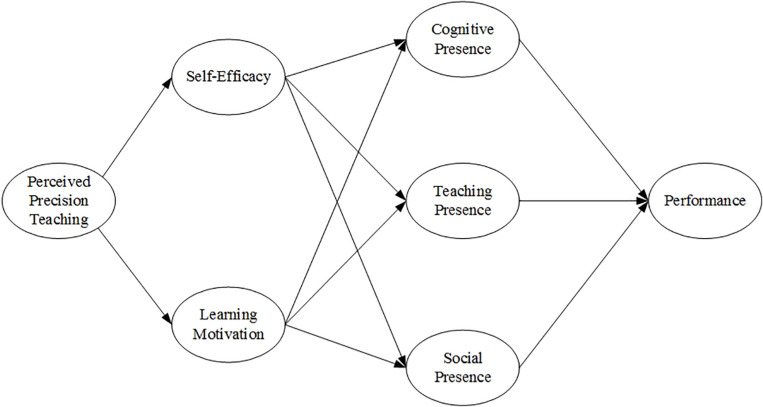
Research model.

Based on the above analysis, the constructed model composed of previous hypotheses is developed, as shown in Figure 2.

## Materials and Methods

### Measurement

We conducted a questionnaire survey to test the above five sets of hypotheses. The questionnaire is adapted from mature scales in the relevant literature and presented in Chinese. We invited a professor of English to translate the scales originally in English into Chinese and another professor of English to check if the translation was appropriate. Then, six students who participated in the course attentively were asked to fill in the questionnaire and inform the researchers of how they understood the questionnaire. In view of the students’ feedback, the two English professors improved the Chinese translation. This was to ensure consistency in content between the Chinese and English scales, as well as the accuracy in the respondents’ understanding. The questionnaire encompasses PPT, self-efficacy (SE), learning motivation (LM), teaching presence (TP), social presence (SP), cognitive presence (CP), and learning performance (PERF). The items and references are detailed in Appendix A. The questionnaire adopted a five-point Likert scale [from “strongly disagree” (1) to “strongly agree” (5)] and the respondents were required to complete the survey anonymously.

### Experiment Setting and Participants

**FIGURE 3:**
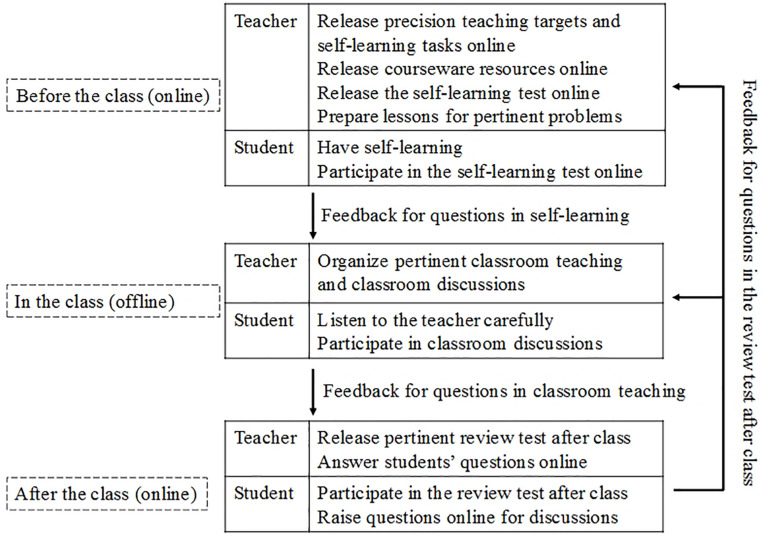
Blended learning process featuring precision teaching.

To test the research model, we provided a blended learning course of “Computer Network Technologies” featuring precision teaching for one term in a university in a large city in China. The participants of our study were 258 students majoring in e-commerce. The learning process is depicted in Figure 3, which was integrated into a blended learning process the four basic steps of precision teaching, that is, pinpoint, record, change, and try again (Benbunan-Fich, 2008; Kubina and Yurich, 2012). At the end of the term, the above-mentioned questionnaire (See Appendix A) was distributed to the participants. Of the 258 students who completed the survey, 256 valid questionnaires remained after the removal of two incomplete ones.

The general information of the respondents is as shown below (Table 1). It can be easily seen that there are more female students than male students, primarily because e-commerce is a management specialty under liberal arts of the school. Most of these students are aged 20–22 years.

**TABLE 1:** Demographic characteristics of students interviewed.

Indicator	Indicator value	Count	Percentage
Gender	Male	101	39.45%
	Female	155	60.55%
Age group	Below 18	0	0.00%
	18–19	5	1.95%
	20–21	100	39.06%
	22–23	145	56.64%
	Above 23	6	2.34%
Total		256	

### Hypothesis Verification

The model (Figure 2) is verified with the partial least squares (PLS) approach of SEM. PLS here refers to an alternative least squares algorithm that expands principal component analysis and typical correlation analysis (Henseler and Sarstedt, 2013). It is composed of two sets of equations that are called the inner model and the outer model, respectively. Among them, the inner model defines the relationship between unobserved variables or latent variables, and the outer counterpart defines the relationship between latent variables and observed indicators. PLS-SEM can predict a relatively complicated model with no need to meet the distribution hypothesis, and for this reason, it is applicable to the handling of non-normal distribution data. This phenomenon is commonplace for business and social science researchers (Fornell and Bookstein, 1982; Hair et al., 2014b; Sarstedt et al., 2014). Accordingly, PLS methods in SmartPLS software are adopted here.

## Results

Analysis and explanation for the PLS model was carried out in two stages: (1) assessment of the measurement model’s reliability and validity and (2) assessment of the structural model.

### Assessment of the Measurement Model

To ensure the fine reliability and validity of the research, a test is conducted to examine the seven dimensions of the structure, including PPT, self-efficacy, learning motivation, social presence, cognitive presence, teaching presence, and learning performance. For each item, the factor loading is above 0.7 (Table 2). Structural convergence validity is assessed in accordance with the estimation results of the measurement model. Reflective measurement is considered reliable when the item’s factor loading over the correlation factor is high (above 0.7) (Fornell and Larcker, 1981). Subsequently, Cronbach’s alpha, Dillon–Goldstein’s rho, composite reliability, and average variance extracted (AVE) are used to test construct reliability and validity (Table 3). For all constructs, Cronbach’s alpha and Dillon–Goldstein’s rho are greater than 0.7, congruent with the acceptable criterion (Nunnally, 1968), and composite reliability is greater than 0.9, far above the recommended value of 0.5 (Chin and Gopal, 1995). Additionally, the AVE value greater than the minimum 0.5 well proves the reliability and validity of the research (Fornell and Larcker, 1981). These indicators demonstrate the feasibility of all constructs for follow-up research.

**TABLE 2:** Outer model estimation.

Latent variable	Manifest variables	Factor Loadings	Outer Weights
Perceived Precision Teaching (PPT)	PPT_1	0.918	0.272
	PPT_2	0.914	0.272
	PPT_3	0.926	0.272
	PPT_4	0.919	0.272
Self-Efficacy (SE)	SE_1	0.814	0.165
	SE_2	0.857	0.183
	SE_3	0.858	0.176
	SE_4	0.860	0.169
	SE_5	0.839	0.184
	SE_6	0.843	0.175
	SE_7	0.741	0.149
Learning Motivation (LM)	LM_1	0.880	0.215
	LM_2	0.905	0.226
	LM_3	0.881	0.220
	LM_4	0.913	0.222
	LM_5	0.914	0.228
Cognitive Presence (CP)	CP_1	0.854	0.170
	CP_2	0.847	0.170
	CP_3	0.834	0.149
	CP_4	0.861	0.169
	CP_5	0.861	0.165
	CP_6	0.882	0.167
	CP_7	0.882	0.172
Social Presence (SP)	SP_1	0.886	0.183
	SP_2	0.882	0.177
	SP_3	0.875	0.183
	SP_4	0.910	0.193
	SP_5	0.892	0.191
	SP_6	0.895	0.196
Teaching Presence (TP)	TP_1	0.919	0.277
	TP_2	0.908	0.258
	TP_3	0.919	0.272
	TP_4	0.911	0.287
Preference (PREF)	PER_1	0.944	0.352
	PER_2	0.942	0.348
	PER_3	0.949	0.357

**TABLE 3:** Construct reliability and validity.

	MVs	Cronbach’s Alpha	Rho_A	Composite Reliability	AVE
PPT	4	0.939	0.939	0.956	0.845
SE	7	0.925	0.928	0.940	0.691
LM	5	0.940	0.941	0.955	0.808
CP	7	0.941	0.942	0.952	0.740
TP	4	0.935	0.936	0.953	0.836
SP	5	0.947	0.948	0.958	0.792
PER	3	0.940	0.940	0.962	0.893

The discriminant validity of the constructs is assessed based on the Fornell and Larcker (1981) criterion. According to Fornell and Larcker (1981), for good discriminant validity, the square root of the AVE of the constructs should be larger than the correlation coefficient between the constructs. All the constructs in the estimated model are in line with this criterion (Fornell and Larcker, 1981; See Table 4).

**TABLE 4:** Fornell and Larcker (1981) criterion.

	PPT	SE	LM	CP	TP	SP	PER
PPT	***0.919***						
SE	0.699	***0.831***					
LM	0.727	0.767	***0.899***				
CP	0.664	0.813	0.816	***0.860***			
TP	0.734	0.679	0.766	0.778	***0.914***		
SP	0.688	0.764	0.799	0.837	0.813	***0.890***	
PER	0.695	0.747	0.774	0.796	0.807	0.802	***0.945***

*The square root of the average variance extracted (AVE) for each construct is denoted in bold and italic, while the inter-construct correlations are shown off-diagonally.*

### Assessment of the Structural Model

**FIGURE 4:**
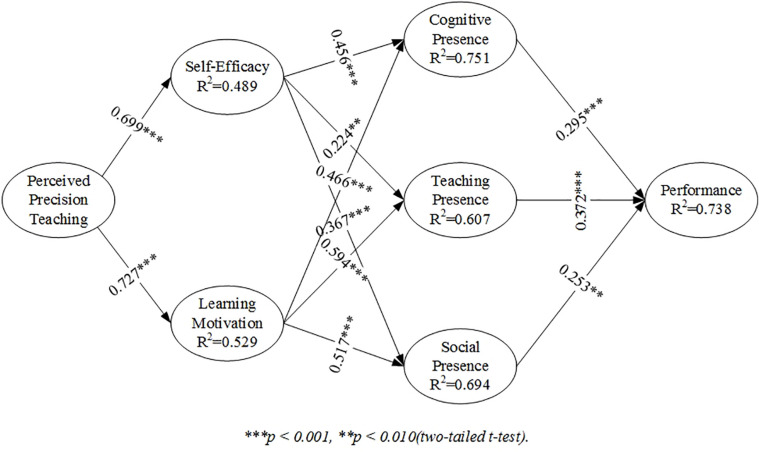
PLS structural model.

Surveyed data executed 5,000 bootstrapping procedure model operations using SmartPLS3. Figure 4 presents the results of the hypothesis verification vividly. The direct and indirect effects of the seven constructs are tested, and the results are as shown in Tables 5, 6.

**TABLE 5:** Structural model path coefficient (1).

	SE		LM		CP	
	Direct	Indirect	Direct	Indirect	Direct	Indirect
PPT	0.699***		0.727***			0.658***
SE					0.456***	
LM					0.466***	
CP						
TP						
SP						

***p < 0.001 (two-tailed t-test).

**TABLE 6:** Structural model path coefficient (2).

	TP		SP		PER	
	Direct	Indirect	Direct	Indirect	Direct	Indirect
PPT		0.589***		0.633***		0.573***
SE	0.224**		0.367***			0.311***
LM	0.594***		0.517***			0.489***
CP					0.295***	
TP					0.372***	
SP					0.253**	

***p < 0.001, **p < 0.010 (two-tailed t-test).

In blended learning, PPT is positively and directly related to self-efficacy (β = 0.699, *p* < 0.001), and learning motivation (β = 0.727, *p* < 0.001), supporting the validity of H1 and H2. PPT is positively and indirectly related to cognitive presence (β = 0.658, *p* < 0.001), teaching presence (β = 0.589, *p* < 0.001), and social presence (β = 0.633, *p* < 0.001), and eventually learning performance (β = 0.573, *p* < 0.001); and self-efficacy is positively and directly related to cognitive presence (β = 0.456, *p* < 0.001), teaching presence (β = 0.224, *p* = 0.001), and social presence (β = 0.367, *p* < 0.001), supporting the validity of H3a, H3b, and H3c, respectively, and therefore attesting that H3 is tenable. Learning motivation is positively and directly related to cognitive presence (β = 0.466, *p* < 0.001), teaching presence (β = 0.594, *p* < 0.001), and social presence (β = 0.517, *p* < 0.001), supporting the validity of H4a, H4b, and H4c, respectively, and therefore attesting that H4 is tenable. Cognitive presence is positively and directly related to learning performance (β = 0.295, *p* < 0.001), teaching presence is positively and directly related to learning performance (β = 0.372, *p* < 0.001), and social presence is positively and directly related to learning performance (β = 0.253, *p* = 0.006), supporting the validity of H5a, H5b, and H5c, respectively and therefore attesting that H5 is tenable.

As PLS path modeling cannot provide a widely accepted global model fitting (Hair et al., 2017b), the research deliberately adopts standardized root mean square residual (SRMR) for model fitting, finding that model SRMR value of 0.042 below 0.08 is acceptable (Hair et al., 2017b).

### Predictive Relevance and Effect Size

In addition to the use of determining the coefficient (R^2^) in assessing the model’s predictive accuracy, cross-validation redundancy (Q^2^) may be also used to determine the predictive relevance of the model (Hair et al., 2014a). The model possesses predictive relevance in the case that the endogenous variable value Q^2^ is greater than 0 (Hair et al., 2017a). Table 7 also suggests that the model has predictive relevance in self-efficacy, learning motivation, cognitive presence, teaching presence, social presence, and learning performance.

**TABLE 7:** Predictive relevance and effect size.

	R^2^	Q^2^	Exogenous variables	Effect size f^2^
SE	0.489	0.333	PPT	0.957
LM	0.529	0.425	PPT	1.123
CP	0.751	0.551	SE	0.344
			LM	0.360
TP	0.607	0.501	SE	0.053
			LM	0.370
SP	0.694	0.543	SE	0.182
			LM	0.359
PER	0.738	0.650	CP	0.090
			TP	0.162
			SP	0.057

Additionally, Table 7 calculates the effect size of each exogenous variable f^2^. According to Hair et al. (2014a), effect size f^2^ indicates the contribution of the exogenous variable to the endogenous variable R^2^, and 0.02, 0.15, and 0.35 suggesting small, medium, and large effects, respectively (Hair et al., 2014a,b). PPT has a large effect on self-efficacy (SE) (*f*^2^ = 0.957) and learning motivation (LM) (*f*^2^ = 1.123); self-efficacy (SE) has a large effect on cognitive presence (CP) (*f*^2^ = 0.344), a small effect on teaching presence (TP) (*f*^2^ = 0.053), and a medium effect on social presence (SP) (*f*^2^ = 0.182); learning motivation has a large effect on cognitive presence (CP) (*f*^2^ = 0.360), teaching presence (TP) (*f*^2^ = 0.370), and social presence (SP) (*f*^2^ = 0.359); cognitive presence (CP) and social presence (SP) have a small effect on learning performance (PER) (*f*^2^ = 0.090, *f*^2^ = 0.057); and teaching presence has a medium effect on learning performance (PER) (*f*^2^ = 0.162).

## Discussion

By analyzing 256 students from a blended learning environment featuring precision teaching at the end of the term, this study sheds light on the relationship between PPT and learning performance in a blended learning environment. Specifically, PPT positively correlates with self-efficacy and learning motivation, while self-efficacy, learning motivation, and COI exerts a serial mediating effect on the relations between PPT and learning performance.

### Perceived Precision Teaching, Self-Efficacy, and Learning Motivation

In this research, we find that PPT is positively related to self-efficacy and learning motivation, with great predictive relevance and effect. It is also indirectly and positively related to learning performance. In a blended learning experience featuring precision teaching, students are requested to learn the online course content by themselves before class and join in the test. Students’ test scores are recorded by the system. In this way, students have already made sufficient preparations and show greater self-efficacy in classroom teaching beforehand. On the part of teachers, they can have pertinent and well-organized teaching interventions for problems discovered in the testing procedure (Kubina and Yurich, 2012). Facts prove that more pertinent teaching easily attracts students’ attention, arouses their interests, and stimulates their self-efficacy and learning motivation.

### Self-Efficacy, Cognitive Presence, Social Presence, Teaching Presence, and Learning Performance

The research reveals that self-efficacy is directly and positively related to cognitive presence, social presence, and teaching presence, with varying predictive relevance and effect size, and is indirectly and positively related to learning performance. In blended learning environments featuring precision teaching, students are asked to preview and join in the test before class, so that they can basically grasp the course content, reinforce self-efficacy, and the willingness to have exchanges with classmates and teachers, and more easily get immersed in the teaching process. These behaviors are all directly and positively related to their learning performance.

### Learning Motivation, Cognitive Presence, Social Presence, Teaching Presence, and Learning Performance

Learning motivation is a key factor for active learning and also a decisive factor for student learning performance and expression (Weiner, 1990; Law et al., 2010; Law and Breznik, 2017; Law and Geng, 2018). Learning motivation may be stimulated by endogenous and exogenous factors, in which endogenous learning motivation is the dominant type in blended learning courses (Lin et al., 2003). Simultaneously, it has been found that learning motivation is directly and positively related to cognitive presence, social presence, and teaching presence, with great predictive relevance and effect, and indirectly and positively related to learning performance. Those students who have strong learning motivation are also inclined to more positively participate in course assignments and group activities. From this perspective, learning motivation promotes learning performance via its positive and direct relation to social presence and teaching presence.

### Serial Mediating Effect of Perceived Precision Teaching on Learning Performance

In this study, it is observed that self-efficacy, learning motivation, and COI (including cognitive presence, teaching presence, and social presence) generate a serial mediating effect between PPT and learning performance, that is, PPT improves learners’ self-efficacy and learning motivation (Chapman et al., 2005; Roberts and Norwich, 2010; Rebecca and Michelle, 2016), while self-efficacy and learning motivation are directly related to COI, and COI is positively related to learning performance (Akyol and Garrison, 2011; Akyol et al., 2011; Kozan and Richardson, 2014).

## Conclusion

This study discusses and verifies the relation between PPT and learning performance in blended learning environments, that is, PPT is positively related to self-efficacy and learning motivation, self-efficacy and learning motivation are positively related to COI (including cognitive presence, teaching presence and social presence), COI is positively related to learning performance, and PPT remotely and positively predicts learning performance.

Throughout the whole process, PPT is not only the starting point but also the most critical link which eventually predicts learning performance. As for the design for blended learning courses featuring precision teaching, the first step is to pinpoint by setting up test questions to check the achievements of learning goals, so that students clearly know their learning goals before participating in online learning, experience pertinent learning, and finally, examine the achievements of learning goals after learning. The second step is record, which illustrates that the online learning system can elaborately record students’ achievements of the learning process, and reflects their problems encountered in the learning process timely and precisely. The third step is change; herein, teachers are expected to precisely grasp the learning conditions and problems via the online learning system before class, prepare lessons based on the data analysis (Bruhn et al., 2020), organize pertinent discussions and classroom teaching during class, and thus ensure that the taught knowledge points exactly to solve what may be puzzling the students. The last step is try again. After class, students must have proper tests in online learning systems to check if their personal learning goals have been achieved yet, and if not, efforts should be made further by the teachers to improve the blended learning effects through pertinent teaching preparations. If precision teaching works, students familiarize themselves with the learning content before class, which in turn, boosts students’ confidence, obviously enhancing their self-efficacy and learning motivation, and motivating them to participate in course learning and class discussions. Pertinent teaching in class further reinforces students’ interests in learning and eventually has a positive impact on learning performance. A good understanding of the flow, content design, and effective implementation of precision teaching is therefore meaningful for obtaining favorable learning performance.

The theoretical significance of this study is that it manifests the relation between PPT in blended learning and learning performance through self-efficacy, learning motivation, and COI, and provides new insight into research on blended learning performance. The meaning of teaching practice is to offer a realistic reference to the development of precision teaching practice in a blended learning environment and present an effective way to improve learning performance. Accompanied by the fast advance of information technology and the sustained growth of teacher-student information attainments, blended learning gains an increasingly wide scope of application, and meanwhile, more and more technical systems create many conveniences to the implementation of precision teaching. It is foreseeable that precision teaching will be applied in various teaching practices in the future.

## Research Limitations and Suggestions for Further Study

Due to the limitation of the course for blended learning featuring precision teaching, our participants are mainly from the same course. It is expected that future research could expand to more blended courses with precision teaching. However, the results are based on an analysis of Chinese students, so the generalizability to students in other countries is yet to be confirmed.

## Data Availability Statement

The raw data supporting the conclusions of this article will be made available by the authors, without undue reservation.

## Ethics Statement

Ethical review and approval was not required for the study on human participants in accordance with the local legislation and institutional requirements. Written informed consent for participation was not required for this study in accordance with the national legislation and the institutional requirements.

## Author Contributions

BY: writing–original draft. C-HY: writing–review and editing. Both authors have read and agreed to the published version of the manuscript.

## Conflict of Interest

The authors declare that the research was conducted in the absence of any commercial or financial relationships that could be construed as a potential conflict of interest.

## Appendix A

**TABLE A1:** Precision teaching survey instrument.

Part One		
Five-point Likert-type scale 1 = strongly disagree, 2 = disagree, 3 = neutral, 4 = agree, 5 = strongly agree.		
List of items label	Initial items	Sources of reference
PPT_1	Teachers have clearly formulated learning requirements and goals in online learning course before class.	Kubina and Yurich, 2012
PPT_2	The online learning system records the scores of my learning process.	
PPT_3	Teachers have pertinent teaching targeted at problems in self-learning online course in classroom teaching.	
PPT_4	Teachers re-examine the teaching effect after classroom teaching.	
SE_1	I can carefully finish self-learning and test on time.	Artino and Mccoach, 2008
SE_2	In classroom teaching, I can carefully participate in course learning.	
SE_3	I can carefully finish the test after class as required.	
SE_4	I can often figure out some countermeasures whenever I have trouble in learning.	
SE_5	I believe that I can finish the learning task even there are interventions.	
SE_6	I can always find a method to have course learning even there are technical problems, such as internet lag.	
SE_7	I can often find several solutions for a learning problem.	
LM_1	When I succeed in finishing the course learning task, my learning motivation will be stimulated.	Law et al., 2019
LM_2	When I succeed in finishing the task, my learning motivation will be stimulated.	
LM_3	I feel glad that I have taken the course.	
LM_4	I am very interested in the course content and it stimulates my initiative in learning the course.	
LM_5	The course can improve my ability and knowledge and stimulate me to learn the course.	
CP_1	I can quickly acquire knowledge from course.	Garrison et al., 2010
CP_2	I can search more course-related information via other means of learning (such as video, discussion and network).	
CP_3	I can find problems existing in the course.	
CP_4	I can connect all knowledge points learned from the course as a whole.	
CP_5	My level of knowledge can be reflected from course learning.	
CP_6	The course allows me to explore more thoughts and integrate my thoughts into the solutions.	
CP_7	The course fosters my excellent thinking ability.	
SP_1	The course gives me a chance to express myself.	Garrison et al., 2010
SP_2	The course gives me a chance to formally interact with my classmates.	
SP_3	The course gives me a chance to non-formally interact with other students.	
SP_4	The course provides enough cooperative learning and discussion activities.	
SP_5	I prefer to attend course activities.	
SP_6	I have a sense of belonging toward the course.	
TP_1	Teachers have clear instructions for the participation of classroom learning activities.	Garrison et al., 2010
TP_2	Learning task allotted by the course has moderate difficulties.	
TP_3	The teaching flow of the course is innovative.	
TP_4	I am satisfied with course information transmission channels and means.	
PERF_1	I have enriched my knowledge through the course.	Law et al., 2019
PERF_2	I have improved my relevant skills through the course.	
PERF_3	I have improved my thinking ability through the course.	

**Part Two**		

Your gender	Male/Female	
Your age group	Below 18;18–19;20–21;22–23; above 23	
